# Vitamin D Receptor Polymorphisms Predispose to Primary Biliary Cirrhosis and Severity of the Disease in Polish Population

**DOI:** 10.1155/2012/408723

**Published:** 2012-05-29

**Authors:** Agnieszka Kempińska-Podhorecka, Ewa Wunsch, Tomasz Jarowicz, Joanna Raszeja-Wyszomirska, Beata Loniewska, Mariusz Kaczmarczyk, Małgorzata Milkiewicz, Piotr Milkiewicz

**Affiliations:** ^1^Medical Biology Laboratory, Pomeranian Medical University, Szczecin, Poland; ^2^Liver Unit and Liver Research Laboratories, Pomeranian Medical University, Szczecin, Poland; ^3^Department of Neonatal Diseases, Pomeranian Medical University, Szczecin, Poland; ^4^Department of Clinical and Molecular Biochemistry, Pomeranian Medical University, Szczecin, Poland

## Abstract

Primary biliary cirrhosis (PBC) is a chronic cholestatic liver condition characterized by the immune-mediated damage of the intrahepatic bile ducts. Polymorphisms of vitamin D receptor (VDR) are considered to contribute to its pathogenesis however their incidence varies in different populations and their potential association with the course of the disease has not been studied. In this paper we investigated the incidence and correlation of three VDR polymorphisms (*BsmI*, *ApaI* or *TaqI*) with various clinical, biochemical, and serological factors in a homogenous group of 143 Caucasian patients with PBC. Control group comprises 306 DNA samples from umbilical cord blood of healthy newborn children. When compared to controls, we observed a significant dominance of the *b* allele in the *BsmI* (OR = 1.69 [1.27–2.24]; *P* = 0.0003) and *t* allele in the *TaqI* (OR = 0.62 [0.47–0.82], *P* = 0.0001) in patients with PBC. Moreover the *BsmI* and *TaqI* polymorphisms were associated with the presence of advanced fibrosis/liver cirrhosis at the diagnosis of PBC. Pairwise linkage disequilibrium (LD) calculations proved that the analyzed SNPs are within an LD block (100% of LDs were *D*'>0.9). Our study showed, for the first time, that the analyzed polymorphisms of VRD may exert an effect on a natural history of PBC.

## 1. Introduction

Primary biliary cirrhosis (PBC) is a chronic cholestatic liver condition [1] which may lead to cirrhosis and liver failure [2, 3]. The presence of highly specific M2 antimitochondrial antibodies (AMAs) is a serological hallmark of the disease [4, 5]. PBC affects mainly women in their 5th and 6th decade of life and is often associated with other autoimmune conditions. Although etiology of PBC remains to be fully elucidated, genetic factors are known to contribute to its pathogenesis [6]. Previous studies suggested major histocompatibility complex (MHC) class II polymorphisms as showing the strongest, mainly protective, association [7]. Four recent genomewide association studies (GWASs) which included (i) North American, (ii) Italian (iii) mostly North American, and (iv) British cohorts, respectively, pointed at a number of genes potentially involved in the pathogenesis of PBC and emphasized the role of IL-12 pathway [8–11].

A fat-soluble secosteroid hormone 1,25-dihydroxyvitamin D3 (1,25(OH)_2_D_3_), apart from its central role in calcium and bone metabolism, possesses immunomodulatory properties and represents an important component of the immune system homeostasis [12]. Many observations suggest that 1,25(OH)_2_D_3_ can influence the risk of various autoimmune conditions, such as rheumatoid arthritis, type 1 diabetes, multiple sclerosis, systemic sclerosis, or inflammatory bowel disease [13–21].

1,25(OH)_2_D_3_ exerts the biological effects through its own nuclear hormone receptor, the vitamin D receptor (VDR), which belongs to the family of transacting transcriptional regulatory factors [22]. VDR is expressed in most tissues and regulates the expression of as many as 500 target genes in the human genome [23]. Recent studies have indicated several polymorphisms in this large gene. Three of them, rs1544410 (*BsmI*), rs7975232 (*ApaI*) (both in intron 8), and rs731236 (*TaqI*; in exon 9), discovered at the 3′ end of the VDR gene, have been analyzed in the past but their effects on VDR function still remain poorly understood [24–26]. Their relationship with PBC has been suggested in various populations including the Japanese, Italian, Chinese, German, or Hungarian [27–30] but not confirmed with previously mentioned GWAS. There are also very little available data about the relationship between these polymorphisms and clinical, laboratory findings and susceptibility to liver cirrhosis in patients with PBC.

In this study we investigated the prevalence of VDR polymorphisms in a homogenous cohort of well-characterized Polish patients with PBC. We also looked at a potential effect of these polymorphisms on the clinical and laboratory features in analyzed cohort of patients.

## 2. Materials and Methods

### 2.1. Patients

One hundred and forty-three patients (20 males, 123 females; median age at diagnosis 55 years, range 28–90 years) were enrolled in this study. All patients met the criteria for the diagnosis of PBC recently introduced by EASL Guidelines [31] according to which PBC can be diagnosed if at least 2 out of the following 3 criteria are fulfilled: elevation of alkaline phosphatase, typical liver histology, and AMA seropositivity. In 102 (71.3%) of patients the diagnosis of PBC was confirmed with liver biopsy out of which in 38 (37.2%) histology showed liver cirrhosis. In total, forty-eight (33.6%) patients had histological/clinical/imaging signs typical for liver cirrhosis.

Umbilical cord blood samples from 306 healthy newborn children served as controls. Data of analyzed subjects are summarized in Table 1. Appropriate informed consent was obtained from each patient included in the study. The study protocol was approved by the ethics committee of Pomeranian Medical University and conforms to the ethical guidelines of the 1975 Declaration of Helsinki (6th revision, 2008).

### 2.2. VDR Genotyping

DNA from peripheral blood mononuclear cells was isolated using the DNeasy Blood & Tissue Kit (Qiagen). Oligonucleotide primers and TaqMan probes for VDR polymorphisms (rs7975232, rs15444410, rs731236) were designed and synthesized by Applied Biosystems (Assay ID: C_28977635_19, C_8716062_10, C_2404008_10, resp.). The fluorescence data were analyzed with allelic discrimination 7500 Software v.2.0.2.

### 2.3. Statistical Analysis

Data are shown as means and standard deviations. All statistical analyses (Chi-Square, odds ratios, confidence intervals) were carried out using StatView software (Carry, NC, US). The genotype and allelic frequencies were compared between patients and controls using Fisher's PLSD test. The analysis of genotype frequency within PBC patients in regards to the clinical characteristics was performed using Fisher's PLSD test. Linkage disequilibrium (LD) for rs7975232, rs15444410, and rs731236 for PBC patients was analyzed with the software Haploview 4.2. *P*  value < 0.05 was consider considered to be statistically significant.

## 3. Results

The following two VDR gene polymorphisms in intron 8 (rs1544410-*BsmI; *rs7975232-*ApaI) *and one in exon 9 (rs731236 = *TaqI) *were analyzed. A significant difference in genotype frequency between patients with PBC and healthy controls at the *BsmI* and the *TaqI *polymorphisms but not at the *ApaI* polymorphism was found. The *X* ^2^ value and the prevalence of each genotype are shown in Table 2. We found a significant association in the frequency of AA (*BB*) genotype at the *BsmI* polymorphism between PBC patients and controls. AA (*BB*) genotype was present less frequently among the patients with PBC (14.0% versus 31.1% in controls; *X* ^2^ = 14,89; *P* = 0.0001). By contrast, at the *TaqI* polymorphisms the CC (*tt*) genotype was represented more frequently (30.2% versus 12.3% in controls; *X* ^2^ = 26,07; *P* < 0,001) and correspondingly, the frequency of TT (*TT*) genotype decreased significantly in patients with PBC in comparison to the controls (16.0% versus 33.2% in controls; *X* ^2^ = 17,39; *P* < 0,001). We found no difference at the polymorphism.

Additionally, the analysis of frequencies of each allele in the three polymorphic sites demonstrated a significant dominance of the *b* allele in the *BsmI* (OR = 1.69 [1.27–2.24]; *P* = 0.0003) and *t* allele in the *TaqI* (OR = 0.62 [0.47–0.82]; *P* = 0.0001) in PBC patients in comparison to controls (Table 3). Moreover, in patients with PBC, the *BsmI *and* TaqI *polymorphisms were associated with the histological features of advanced fibrosis defined as stage III and IV on histology and cirrhosis (stage IV on histology) (Table 4). Both laboratory markers of the disease severity and AMAs failed to have any association to analyzed polymorphisms (Table 4).

**Figure 1:**
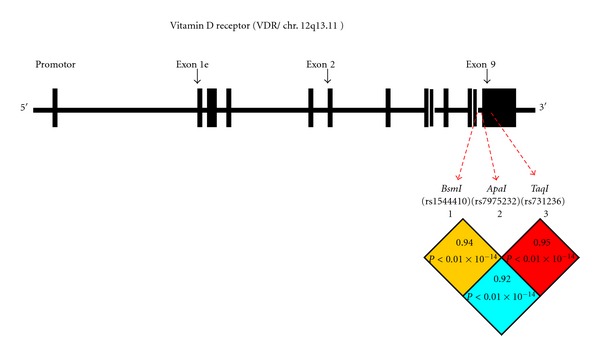
Pairwise linkage disequilibrium (LD) pattern of the VDR gene SNPs: Rs15444410, rs7975232, rs731236. The location of each examined SNP along the chromosome is indicated. Each square plots the level of D' values between the pair of SNPs.

Pairwise linkage disequilibrium (LD) calculations confirmed that the analyzed SNPs are within a LD block (100% of LDs were *D'* > 0.9) (Figure 1).

## 4. Discussion

In this study we have analyzed the association between *BsmI, ApaI, *and* TaqI* polymorphisms and the susceptibility for PBC and severity of the disease in a homogenous group of Polish patients. We have found that *BsmI* polymorphism occurred significantly more commonly in patients with PBC than in controls. These findings are consistent with the ones by Vogel et al. [32] and Fan et al. [33], whereas other studies have found a protective association of the “*b*” allele [27, 34]. Moreover we have shown the high prevalence of *TaqI* polymorphism in patients with PBC, what is again in agreement with the observation by Vogel et al. [32]. In our study a strong association with PBC was demonstrated with “*t*” allele as well as “*tt*” genotype, but in the study by Vogel “Tt” genotype was predictive for PBC. Additionally, the study from Tanaka et al. indicated the high prevalence of *ApaI* in PBC [27], but our study did not show such association.

One may say that in the era of genomewide association studies which involve very large cohorts of subjects analyses aimed at looking at selected polymorphisms in smaller groups have lost their significance. In the context of PBC, four GWASs have been performed and included homogenous groups from Northern American, Italian, mostly Northern American, and British patients, respectively. The first one suggested the role of polymorphisms of gene encoding IL-12 alpha and beta chains [8]. Further GWASs have replicated this finding but also identified new loci associated with antigen-presenting cells and CD4 interactions. Most recent British GWAS identified 12 new susceptibility loci [11].

These results clearly show that each new GWAS adds new susceptibility loci identified in analyzed populations. Thus the fact that VDR polymorphisms were not found in already performed GWAS does not definitely preclude their role in the susceptibility to PBC.

In addition, we have looked at the potential correlation between polymorphisms of VDR gene and clinical and laboratory findings in analyzed patients. We noticed that the polymorphisms of *BsmI* and *TaqI* could be associated with the susceptibility to liver cirrhosis or advanced fibrosis in these subjects which is a new finding. However we found no significant correlations between the cholestasis or the prevalence of autoantibodies and the presence of analyzed polymorphisms.

Little is known on how the genotype variety of VDR gene is linked to clinical course of the disease as previous studies focused mostly on the prevalences of each polymorphisms in PBC. It is possible that the allelic variety of *BsmI* and *TaqI* polymorphisms somehow predisposes to more severe liver injury, independently of the biochemical measurements of cholestasis or autoimmune process. Other studies suggested that VDR polymorphisms can contribute to the development of osteoporosis in patients with PBC [35]; however on the grounds of recent evidence, it is more likely that the cholestasis itself is the main factor of hepatic osteodystrophy, and the indentified polymorphisms play little or no role in the pathogenesis of this complication in patients with PBC [36].

Interpretation of the significance of polymorphisms in the VDR gene is hindered by the fact that the functional effects of allelic variations are poorly understood. Indeed, VDR polymorphisms are considered to be associated with an increased risk of several autoimmune diseases, but so far no correlation has been identified with functional phenotypes [27]. It can be explained by the fact that the *TaqI* polymorphism results in a silent mutation in exon 9 and the location of the *BsmI *and* ApaI *is intronic, thus probably these polymorphisms are anonymous [37]. Thus it could be speculated that *BsmI* and *TaqI* are genetic markers of the presence of other, so far unknown functional sequence variations nearby in the VDR gene which is in linkage disequilibrium with identified alleles [27, 32]. Because the linkage disequilibrium can vary through populations, in different studies the same polymorphisms can be associated with inverse relationships to disease susceptibility. Additionally, the pathogenesis of PBC is for sure multifactorial, influenced by several environmental and genetic interactions and the analysis of one or few genetic factors in different populations may be insufficient to explain observed variables.

The immunomodulatory properties of 1,25(OH)_2_D_3_ may be one of possible explanations on how VDR polymorphisms can contribute to the pathogenesis of PBC. 1,25(OH)_2_D_3_ activates monocytes and macrophages and acts as a positive regulator of the development of Th2 cells and production of anti-inflammatory interleukin (IL)-4 and transforming growth factor *β* [38, 39]. Moreover, VDR agonists inhibit lymphocyte Th1 cell proliferation and production of a number of proinflammatory cytokines (i.e., IL-1, IL-2, IL-6, IL-12, interferon *γ*, tumor necrosis factor *α*, and *β*) [40–42]. PBC is considered a Th1-mediated liver disease [43]; therefore, reduced activity of 1,25(OH)_2_D_3_-dependent signaling pathways caused by the VDR polymorphisms might skew the immune response to the Th1 pathway, contributing to the development of PBC [27].

Other mechanism, in which VDR polymorphisms can be involved in the pathogenesis of PBC, is a role of VDR as a receptor for a lithocholic acid (LCA) as well as an endocrine receptor for vitamin D signaling. LCA is a secondary bile acid produced by intestinal bacteria from primary bile acids that may escape reabsorption in the intestine and can accumulate to high levels in the enterohepatic circulation of some subjects [44]. LCA is hepatotoxic and has been considered to be involved in the pathogenesis of cholesterol gallstone disease and colon cancer [45, 46]. VDR is expressed in biliary epithelial cells [47], where it is considered to act as a sensor for toxic secondary bile acids and to induce their elimination through a xenobiotic metabolism pathway. It can be speculated that VDR polymorphisms may somehow disturb the effective biliary elimination of LCA contributing in this way to the biliary damage and development of PBC. The investigation of VDR-regulated LCA metabolism will be helpful in understanding the potential role of the secondary bile acids in PBC pathogenesis.

In summary, in this present study we have shown the association of* BsmI *and* TaqI *polymorphisms of VDR with the susceptibility to PBC in a cohort of Polish patients. Also we have shown that these patients have an increased risk of liver cirrhosis and advanced fibrosis which is a novel finding of this study. Further studies focused not only on the identification of risk polymorphisms involved in development of PBC but also on the mechanism in which they lead to development and the course of the disease are warranted.

## Figures and Tables

**Table 1:** Demographic data of analyzed subjects.

Feature	PBC (*n* = 143)	Control group (*n* = 306)
Age (median; range)	55 (28–90)	NA
Gender (M/F)	20/123	NA
Biopsy-confirmed cirrhosis (Y/N)	38/64	NA
AMA (pos/neg)	118/25	NA
ALT (median; range) IU/l (*N*—3–30)	47 (10–987)	NA
AP (median; range ), IU/l (*N*—40–120)	286 ( 37–1344)	NA
GGT (median; range), IU/l (*N*—3–30)	177 ( 11–1932)	NA
Bilirubin (median; range), mg/dl (*N*—0.2–1.0)	0.9 (0.2–45.0)	NA
Albumin (median; range), g/dl (*N*—3.8–4.4)	4.0 (2.1–5.8)	NA
INR (median; range) (*N*—0.8–1.2)	1.0 (0.8–2.3)	NA
Cholesterol (median; range ), mg/dl (*N* < 200)	217 (50–1096)	NA
Triglycerides (median; range), mg/dl (*N* < 150)	105 (47–681)	NA

**Table 2:** Distribution of VDR polymorphisms (rs1544410, rs7975232, rs731236) in PBC patients and controls subjects.

Genotype	PBC (%) (*n* = 143)	Controls (%) (*n* = 306)	*χ* ^2^	*P*
rs1544410 *(BsmI) *				

AA *(BB) *	20 (14.0%)	95 (31.1%)	**14.89**	**0.0001**
GA *(bB) *	75 (52.4%)	135 (44.1%)	2.49	0.096
GG *(bb) *	48 (33.6%)	76 (24.8%)	3.71	0.054

rs7975232 *(ApaI) *				

TT *(AA) *	40 (28%)	75 (24.9%)	0.61	0.43
GT *(aA) *	80 (56%)	161 (53.5%)	0.43	0.51
GG *(aa) *	23 (16%)	65 (21.6%)	1.64	0.41

rs731236 *(TaqI) *				

TT *(TT) *	20 (16%)	100 (33.2%)	**17.39**	***<0*.*0001 ***
TC *(Tt) *	77 (53.8%)	140 (54.5%)	2.55	*0*.*26 *
CC *(tt) *	46 (30.2%)	37 (12.3%)	**26.07**	***<0*.*0001 ***

**Table 3:** Allele association for VDR in patients with PBC and control subjects.

SNP	Allele	PBC (%) (*n* = 143)	Controls (%) (*n* = 306)	OR (95% CI)	*P*
rs1544410 * (BsmI) *	G/A *(b/B) *	115 (40.2%)/171 (59.8%)	320 (53.2%)/282 (46.8%)	1.69 (1.27–2.24)	**0.0003**
rs7975232 * (ApaI) *	G/T * (a/A) *	160 (56%)/126 (44%)	311 (51.7%)/291 (48.3%)	0.84 (0.63–1.2)	0.25
rs731236 * (TaqI) *	T/C * (T/t) *	117 (41%)/169 (59%)	364 (60.5%)/238 (39.5%)	0.62 (0.47–0.82)	**0.0001**

PBC: primary biliary cirrhosis; OR: odds ratio; CI: confidence interval.

**Table:** (a)

*ApaI* (rs7975232)						
	AA *(TT) *(*n* = 40)	aA *(GT) *(*n* = 80)	aa *(GG) *(*n* = 23)	*P* * (AA versus Aa)*	*P* * (aa versus Aa)*	*P* *(aa versus AA) *
Gender (F/M)	36/4	67/13	20/3	0.4	0.9	0.7
Cirrhosis at Lbx (N/Y)	20/8	34/21	10/9	0.5	0.6	0.2
Advanced fibrosis stage III/IV on histology (N/Y)	15/11	28/27	8/10	0.6	0.3	0.5
Age of diagnosis (years)	55.0 ± 1.7	55.7 ± 1.5	58 ± 2.2	0.8	0.4	0.3
AST (IU/mL)	60.0 ± 7.9	81.5 ± 14.8	104.5 ± 40.4	0.4	0.4	0.2
ALT (IU/mL)	57.0 ± 8.0	82.1 ± 14.4	84.7 ± 21.4	0.2	0.9	0.3
AP (IU/mL)	226.9 ± 31.5	288 ± 25.9	371.3 ± 64.5	0.2	0.1	**0.02**
GGT (IU/mL)	195.7 ± 32.0	279.0 ± 36.6	280.8 ± 44.5	0.1	0.9	0.2
Bilirubin (mg/dL)	4.0 ± 1.7	4.2 ± 0.9	2.3 ± 0.9	0.9	0.3	0.4
Albumin (g/dL)	3.6 ± 9.7	3.8 ± 0.1	3.9 ± 0.1	0.1	0.9	0.2
INR	1.1 ± 0.04	1.1 ± 0.03	1.1 ± 0.03	0.1	0.1	0.9
Cholesterol (mg/dL)	220.6 ± 14.3	241.4 ± 18.5	227.3 ± 146	0.4	0.7	0.8
TG (mg/dL)	142.4 ± 22.9	120.6 ± 6.8	125.7 ± 18	0.2	0.8	0.5
Sp100 Ab	34/4	62/16	17/5	0.3	0.8	0.3
Gp210 Ab	31/7	67/11	21/1	0.6	0.3	0.2
AMA (Y/N)	31/9	68/12	19/4	0.3	0.7	0.7

**Table:** (b)

*BsmI *(rs15444410)						
	bb (GG) (*n* = 48)	bB (GA) (*n* = 75)	BB (AA) (*n* = 20)	*P * * (bb versus Bb)*	*P * * (BB versus B)*	*P * * (bb versus BB) *
Gender (F/M)	40/8	66/9	17/3	0.6	0.7	0.9
Cirrhosis at Lbx (N/Y)	17/22	38/13	9/3	**0.004**	0.9	0.1
Advanced fibrosis stage III/IV on histology (N/Y)	15/23	30/20	6/5	0.1	0.7	0.5
Age of diagnosis (years)	57.0 ± 1.6	54.4 ± 1.4	59.8 ± 3.1	0.2	0.1	0.4
AST (IU/mL)	110.0 ± 29.2	63.0 ± 5.6	63.1 ± 13.3	0.05	0.9	0.2
ALT (IU/mL)	91.9 ± 22.3	68.6 ± 9.0	62.7 ± 13.5	0.2	0.8	0.3
AP (IU/mL)	321.8 ± 35.3	283.2 ± 29.3	195.3 ± 26.4	0.4	0.2	0.06
GGT (IU/mL)	312.0 ± 48.3	240.1 ± 29.6	170.4 ± 35.6	0.2	0.3	0.7
Bilirubin (mg/dL)	3.3 ± 1.0	4.4 ± 1.1	2.8 ± 2.0	0.5	0.5	0.8
Albumin (g/dL)	3.9 ± 0.1	8.4 ± 4.6	3.9 ± 0.2	0.4	0.6	0.9
INR	1.1 ± 0.02	1.1 ± 0.03	1.1 ± 0.1	0.2	0.4	0.9
Cholesterol (mg/dL)	233.6 ± 18.6	239.5 ± 17.1	206.8 ± 21.2	0.8	0.4	0.5
TG (mg/dL)	126.2 ± 9.9	133.9 ± 13.3	103 ± 10.4	0.6	0.2	0.4
Sp100 Ab	35/12	63/10	15/3	0.1	0.7	0.5
Gp210 Ab	41/6	62/11	16/2	0.8	0.9	0.9
AMA (Y/N)	40/8	63/12	15/5	>0.9	0.3	0.5

**Table:** (c)

*TaqI* (rs731236)						
	TT (TT) (*n* = 20)	Tt (TC) (*n* = 77)	Tt (CC) (*n* = 46)	*P * * (TT versus tT)*	*P * * (tt versus tT)*	*P* * (tt versus TT) *
Gender (F/M)	37/9	69/8	17/3	0.2	0.7	0.7
Cirrhosis at Lbx (N/Y)	15/9	40/13	9/3	0.3	>0.9	0.7
Advanced fibrosis Stage III/IV on histology (N/Y)	13/23	32/20	6/5	0.03	0.7	0.3
Age of diagnosis (years)	57 ± 1.6	54 ± 1.4	60 ± 3	0.2	0.1	0.4
AST (IU/mL)	114.3 ± 30.4	61.6 ± 5.5	63.4 ± 13.2	0.03	0.9	0.2
ALT (IU/mL)	94.2 ± 23.2	67.8 ± 8.8	63.3 ± 13.4	0.2	0.9	0.3
AP (IU/mL)	322.7 ± .36.4	283.6 ± 28.7	195.5 ± 26.4	0.4	0.2	0.06
GGT (IU/mL)	309.3 ± 50.2	243.1 ± 29.2	173.4 ± 35.1	0.2	0.4	0.09
Bilirubin (mg/dL)	3.9 ± 1.1	4.1 ± 1.0	2.8 ± 1.9	0.9	0.6	0.6
Albumin (g/dL)	3.8 ± 0.1	8.3 ± 4.5	3.9 ± 0.2	0.4	0.6	0.9
INR	1.1 ± 0.03	1.1 ± 0.03	1.1 ± 0.1	0.6	0.5	0.7
Cholesterol (mg/dL)	234.7 ± 19.6	239.2 ± 16.5	204.4 ± 21.2	0.8	0.3	0.4
TG (mg/dL)	125.5 ± 10.3	133.9 ± 12.8	103.8 ± 10.8	0.6	0.2	0.4
Sp100 Ab	34/11	64/11	15/3	0.2	0.9	0.7
Gp210 Ab	39/6	64/11	16/2	0.9	0.9	0.9
AMA (Y/N)	40/6	62/15	16/4	0.5	>0.9	0.5

Lbx: liver biopsy; AspAT: aspartate aminotransferase; AlAT: alanine aminotransferase; AP: alkaline phosphatase; GGT: gamma-glutamyl transferase; INR: international normalized ratio; TG: triglycerides, AMA: antimitochondrial antibodies.

## References

[1] Neuberger J (1997). Primary biliary cirrhosis. *The Lancet*.

[2] Kaplan MM, Gershwin ME (2005). Primary biliary cirrhosis. *The New England Journal of Medicine*.

[3] Poupon RE, Lindor KD, Parés A, Chazouillères O, Poupon R, Heathcote EJ (2003). Combined analysis of the effect of treatment with ursodeoxycholic acid on histologic progression in primary biliary cirrhosis. *Journal of Hepatology*.

[4] Gershwin ME, Mackay IR, Sturgess A, Coppel RL (1987). Identification and specificity of a cDNA encoding the 70 KD mitochondrial antigen recognized in primary biliary cirrhosis. *Journal of Immunology*.

[5] Milkiewicz P, Buwaneswaran H, Coltescu C, Shums Z, Norman GL, Heathcote EJ (2009). Value of autoantibody analysis in the differential diagnosis of chronic cholestatic liver disease. *Clinical Gastroenterology and Hepatology*.

[6] Selmi C, Mayo MJ, Bach N (2004). Primary biliary cirrhosis in monozygotic and dizygotic twins: genetics, epigenetics, and environment. *Gastroenterology*.

[7] Invernizzi P, Selmi C, Poli F (2008). Human leukocyte antigen polymorphisms in Italian primary biliary cirrhosis: a multicenter study of 664 patients and 1992 healthy controls. *Hepatology*.

[8] Hirschfield GM, Liu X, Xu C (2009). Primary biliary cirrhosis associated with HLA, IL12A, and IL12RB2 variants. *The New England Journal of Medicine*.

[9] Hirschfield GM, Liu X, Han Y (2010). Variants at IRF5-TNPO3, 17q12-21 and MMEL1 are associated with primary biliary cirrhosis. *Nature Genetics*.

[10] Liu X, Invernizzi P, Lu Y (2010). Genome-wide meta-analyses identify three loci associated with primary biliary cirrhosis. *Nature Genetics*.

[11] Mells GF, Floyd JA, Morley KI (2011). Genome-wide association study identifies 12 new susceptibility loci for primary biliary cirrhosis. *Nature genetics*.

[12] Peelen E, Knippenberg S, Muris AH (2011). Effects of vitamin D on the peripheral adaptive immune system: a review. *Autoimmunity Reviews*.

[13] Cutolo M, Pizzorni C, Sulli A (2011). Vitamin D endocrine system involvement in autoimmune rheumatic diseases. *Autoimmunity Reviews*.

[14] Arnson Y, Amital H, Agmon-Levin N (2011). Serum 25-OH vitamin D concentrations are linked with various clinical aspects in patients with systemic sclerosis: a retrospective cohort study and review of the literature. *Autoimmunity Reviews*.

[15] Pelajo CF, Lopez-Benitez JM, Miller LC (2010). Vitamin D and autoimmune rheumatologic disorders. *Autoimmunity Reviews*.

[16] Amital H, Szekanecz Z, Szücs G (2010). Serum concentrations of 25-OH vitamin D in patients with systemic lupus erythematosus (SLE) are inversely related to disease activity: is it time to routinely supplement patients with SLE with vitamin D?. *Annals of the Rheumatic Diseases*.

[17] Hajas A, Sandor J, Csathy L (2011). Vitamin D insufficiency in a large MCTD population. *Autoimmunity Reviews*.

[18] Souberbielle JC, Body JJ, Lappe JM (2010). Vitamin D and musculoskeletal health, cardiovascular disease, autoimmunity and cancer: recommendations for clinical practice. *Autoimmunity Reviews*.

[19] Adorini L, Penna G (2008). Control of autoimmune diseases by the vitamin D endocrine system. *Nature Clinical Practice Rheumatology*.

[20] Holick MF (2007). Vitamin D deficiency. *The New England Journal of Medicine*.

[21] Grant WB (2006). Epidemiology of disease risks in relation to vitamin D insufficiency. *Progress in Biophysics and Molecular Biology*.

[22] Norman AW (2008). From vitamin D to hormone D: fundamentals of the vitamin D endocrine system essential for good health. *American Journal of Clinical Nutrition*.

[23] Carlberg C (2003). Current understanding of the function of the nuclear vitamin D receptor in response to its natural and synthetic ligands. *Recent Results in Cancer Research*.

[24] Morrison NA, Qi JC, Tokita A (1994). Prediction of bone density from vitamin D receptor alleles. *Nature*.

[25] Morrison NA, Yeoman R, Kelly PJ, Eisman JA (1992). Contribution of trans-acting factor alleles to normal physiological variability: vitamin D receptor gene polymorphisms and circulating osteocalcin. *Proceedings of the National Academy of Sciences of the United States of America*.

[26] Faraco JH, Morrison NA, Baker A, Shine J, Frossard PM (1989). ApaI dimorphism at the human vitamin D receptor gene locus. *Nucleic Acids Research*.

[27] Tanaka A, Nezu S, Uegaki S (2009). Vitamin D receptor polymorphisms are associated with increased susceptibility to primary biliary cirrhosis in Japanese and Italian populations. *Journal of Hepatology*.

[28] Fan L, Tu X, Zhu Y (2005). Genetic association of vitamin D receptor polymorphisms with autoimmune hepatitis and primary biliary cirrhosis in the Chinese. *Journal of Gastroenterology and Hepatology*.

[29] Vogel A, Strassburg CP, Manns MP (2002). Genetic association of vitamin D receptor polymorphisms with primary biliary cirrhosis and autoimmune hepatitis. *Hepatology*.

[30] Halmos B, Szalay F, Cserniczky T (2000). Association of primary biliary cirrhosis with vitamin D receptor BsmI genotype polymorphism in a Hungarian population. *Digestive Diseases and Sciences*.

[31] European Association for the Study of the Liver (2009). EASL clinical practice guidelines: management of cholestatic liver diseases. *Journal of Hepatology*.

[32] Vogel A, Strassburg CP, Manns MP (2002). Genetic association of vitamin D receptor polymorphisms with primary biliary cirrhosis and autoimmune hepatitis. *Hepatology*.

[33] Fan L, Tu X, Zhu Y (2005). Genetic association of vitamin D receptor polymorphisms with autoimmune hepatitis and primary biliary cirrhosis in the Chinese. *Journal of Gastroenterology and Hepatology*.

[34] Halmos B, Szalay F, Cserniczky T (2000). Association of primary biliary cirrhosis with vitamin D receptor BsmI genotype polymorphism in a Hungarian population. *Digestive Diseases and Sciences*.

[35] Springer JE, Cole DE, Rubin LA (2000). Vitamin D-receptor genotypes as independent genetic predictors of decreased bone mineral density in primary biliary cirrhosis. *Gastroenterology*.

[36] Parés A, Guañabens N, Rodés J (2005). Gene polymorphisms as predictors of decreased bone mineral density and osteoporosis in primary biliary cirrhosis. *European Journal of Gastroenterology and Hepatology*.

[37] Uitterlinden AG, Fang Y, van Meurs JBJ, Pols HAP, Van Leeuwen JPTM (2004). Genetics and biology of vitamin D receptor polymorphisms. *Gene*.

[38] Cantorna MT, Woodward WD, Hayes CE, DeLuca HF (1998). 1,25-dihydroxyvitamin D3 is a positive regulator for the two anti- encephalitogenic cytokines TGF-*β*1 and IL-4. *Journal of Immunology*.

[39] Boonstra A, Barrat FJ, Crain C, Heath VL, Savelkoul HFJ, O’Garra A (2001). 1*α*,25-dihydroxyvitamin D3 has a direct effect on naive CD4+ T cells to enhance the development of Th2 cells. *Journal of Immunology*.

[40] Lemire JM, Archer DC, Beck L, Spiegelberg HL (1995). Immunosuppressive actions of 1,25-dihydroxyvitamin D3: preferential inhibition of Th1 functions. *Journal of Nutrition*.

[41] Willheim M, Thien R, Schrattbauer K (1999). Regulatory effects of 1*α*,25-dihydroxyvitamin D3 on the cytokine production of human peripheral blood lymphocytes. *Journal of Clinical Endocrinology and Metabolism*.

[42] Cippitelli M, Santoni A (1998). Vitamin D3: a transcriptional modulator of the interferon-*γ* gene. *European Journal of Immunology*.

[43] Harada K, Nakanuma Y (2007). Biliary innate immunity and cholangiopathy. *Hepatology Research*.

[44] Ridlon JM, Kang DJ, Hylemon PB (2006). Bile salt biotransformations by human intestinal bacteria. *Journal of Lipid Research*.

[45] Makishima M, Lu TT, Xie W (2002). Vitamin D receptor as an intestinal bile acid sensor. *Science*.

[46] McGarr SE, Ridlon JM, Hylemon PB (2005). Diet, anaerobic bacterial metabolism, and colon cancer: a review of the literature. *Journal of Clinical Gastroenterology*.

[47] Gascon-Barré M, Demers C, Mirshahi A, Néron S, Zalzal S, Nanci A (2003). The normal liver harbors the vitamin D nuclear receptor in nonparenchymal and biliary epithelial cells. *Hepatology*.

